# Adipocytokines as Predictors of Metabolic Dysfunction-Associated Steatotic Liver Disease (MASLD) Development in Type 2 Diabetes Mellitus Patients

**DOI:** 10.7759/cureus.55673

**Published:** 2024-03-06

**Authors:** Almir Fajkić, Rijad Jahić, Almira Hadžović-Džuvo, Orhan Lepara

**Affiliations:** 1 Department of Pathophysiology, University of Sarajevo Faculty of Medicine, Sarajevo, BIH; 2 Department of Internal Medicine, General Hospital “Prim. Dr. Abdulah Nakas”, Sarajevo, BIH; 3 Department of Human Physiology, University of Sarajevo Faculty of Medicine, Sarajevo, BIH

**Keywords:** metabolic dysfunction-associated steatotic liver disease (masld), type 2 diabetes mellitus (t2dm), liver steatosis, resistin, adiponectin

## Abstract

Background: Metabolic dysfunction-associated steatotic liver disease (MASLD) is a common chronic liver condition. Due to pathophysiological processes, MASLD's relation to type 2 diabetes mellitus (T2DM) is still unclear, especially when the role of adipocytokines is taken into consideration.

Objective: This study aims to examine the potential predictive value of adiponectin and resistin for MASLD in T2DM.

Patients and methods: In a two-year study, 71 T2DM patients were categorized into MASLD-T2DM and non-MASLD-T2DM groups according to MASLD development. Serum samples were tested for resistin, adiponectin, high-density lipoprotein cholesterol, fasting glucose, and triglycerides. An appropriate equation is used to calculate the adiponectin/resistin (A/R) index. The optimal cut-off values for differentiating MASLD patients from non-MASLD patients were determined using receiver operating characteristic (ROC) curves and the corresponding areas under the curve (AUC). To predict the onset of MASLD in patients with T2DM, a logistic regression analysis was performed.

Results: There were significant differences in adiponectin (p<0.001), resistin (p<0.001), and A/R index (p<0.001) between T2DM individuals with and without MASLD. The ROC curve for resistin produced an AUC of 0.997 (p<0.001) with a sensitivity of 96.1% and a specificity of 100% for the cut-off point of 253.15. Adiponectin (OR, 0.054; 95% CI, 0.011-0.268; p<0.001) and resistin (OR, 1.745; 95% CI, 1.195-2,548; p=0.004) were found to be independent predictors for MASLD by logistic regression analysis.

Conclusion: This study confirms the potential of adiponectin and resistin as predictors of MASLD development in T2DM.

## Introduction

Metabolic dysfunction-associated steatotic liver disease (MASLD), previously known as non-alcoholic fatty liver disease (NAFLD), is becoming one of the most common causes of chronic liver disorders. Various liver diseases are included in MASLD, ranging from basic steatosis to metabolic dysfunction-associated steatohepatitis, accompanied by fibrosis, inflammation, and hepatocyte destruction [1].

Since there is no clear pathophysiological explanation for what has been called for more than 20 years "non-alcoholic fatty liver disease," terms like "alcoholic" and "fatty" have long been used loosely to describe the hepatic manifestation of a systemic metabolic disorder that is primarily associated with cardiovascular consequences. It has led to recent changes in the nomenclature of NAFLD [2].

Patients with at least one of the five cardiometabolic risk factors - hypertension, T2DM, obesity, hypertriglyceridemia, and low- and high-density cholesterol - as well as hepatic steatosis are classified as having MASLD. An estimated 47 cases of MASLD occur globally for every 1000 people in the population; this prevalence is expected to increase to 63% by the end of 2030 [3,4].

Because most MASLD patients are asymptomatic and are first detected by standard blood tests that reveal high liver enzymes, diagnosing MASLD can be difficult. Some patients can have normal liver enzymes and remain undiagnosed [2,5].

This chronic, progressive condition is a type of steatosis that is caused by factors other than excessive alcohol use. The exact cause is not entirely understood, but it often occurs with other metabolic disorders such as obesity and diabetes, and that can be the answer for the increasing prevalence of MASLD, which parallels the increasing prevalence of obesity and obesity-related diseases [6].

T2DM patients represent an important target group for the detection of MASLD, which can even progress to liver fibrosis with the development of more severe comorbidities. In both conditions, a possible key factor could be the presence of insulin resistance (IR) and adipose tissue disorders that are linked to the pathophysiology of “metabolic dysfunction,” liver disease, and the development of cardiovascular and metabolic complications [7].

Visceral adipose tissue functions as an endocrine organ, releasing various hormones and signaling molecules, while adipocytokines play a crucial role in the etiology and progression of MASLD. Adipose tissue is a reservoir of fatty acids, and when storage ability is overwhelmed, the endocrine functions of adipose tissues are altered. The ensuing accumulation of ectopic fat leads to lipotoxicity, which promotes tissue low-grade inflammation and IR in the liver [8,9]. In addition, patients with MASLD have different blood levels of adipocytokines, and new studies have shown how important adipokines are in controlling fibrosis, inflammation, and IR in MASLD [10,11].

Adiponectin, the only adipocytokine with down-regulated levels in obesity, shows an inverse correlation with body mass index and seems essential in reducing body fat. Additionally, it increases insulin sensitivity, lowers hepatic inflammation, and prevents lipid buildup in the liver by stimulating hepatocytes to β-oxidize free fatty acids. Additionally, some research studies have shown a negative correlation between the degree of inflammation, hepatic steatosis, and adiponectin levels [12,13].

In contrast to adiponectin, resistin induces hepatic IR, exerts proinflammatory effects, is implicated in hepatic lipogenesis, and triggers liver fibrogenesis. In patients with diagnosed fatty liver disease, serum resistin levels correlate with the severity of steatosis, inflammation, and fibrosis [14].

## Materials and methods

Study sample and design

This two-year study initially included 85 T2DM patients of both sexes without MASLD, selected randomly in family medicine centers in Sarajevo Canton. After 24 months of observation, 71 patients met the research criteria, and their data were taken for statistical analysis. All the patients provided written informed consent before study entry, and the study received approval from the Ethical Committee of the Faculty of Medicine at the University of Sarajevo (approval number: 02-3-4-4493/2). The study is conducted according to the principles outlined in the Declaration of Helsinki concerning the rights of patients participating in biomedical research (Revision 2013).

At the end of the study, individuals with T2DM who met the research criteria were divided into two groups: (a) the MASLD-T2DM group (patients who met MASLD criteria during the study (n=47)) and (b) the non-MASLD-T2DM group (patients who did not meet MASLD criteria during the research (n=24)).

Inclusion and exclusion criteria

The inclusion criteria for the study were T2DM patients of both genders aged between 40 and 60 years, patients with a T2DM duration of less than five years, patients with normal levels of liver enzymes, and a BMI value of less than 25 kg/m^2^.

T2DM patients with the following conditions were excluded from the study: patients with chronic liver diseases, cardiovascular and kidney diseases inducing liver congestion, serious infections, and malignancy, heavy alcohol consumption, ascites, elevated aminotransferase levels more than five times the upper normal limit, or taking any drugs known to cause disturbance of liver function.

Definition of MASLD

The presence of MASLD was determined based on the presence of liver steatosis detected by imaging plus at least one of the five following features [1]: (a) BMI ≥25 kg/m^2^ or waist circumference >94 cm in men and >80 cm in women, (b) fasting serum glucose ≥5.6 mmol/L or HbA1c ≥5.7%, (c) blood pressure ≥130/85 mmHg, (d) triglycerides ≥1.70 mmol/L, and (e) HDL cholesterol <1.0 mmol/L for men and <1.3 mmol/L for women.

Data collection

At the commencement and conclusion of the research period, comprehensive data were collected from all T2DM patients, including medical history, height, and weight measured to determine BMI (kg/m^2^), waist circumference, and blood pressure measurement.

After taking the medical history and physical examination, blood was taken for laboratory tests by puncture of the cubital vein. Within half an hour of blood collection, the serum is separated from the blood by centrifugation. Until the examination, the samples were stored at -20°C.

The patients had the following laboratory tests using standard enzymatic colorimetric techniques (Dimension RxL Max, Dade Behring, Germany) at the start of the study and 24 months later: fasting blood glucose, triglycerides, and HDL cholesterol. An immunoturbidimetric method (Roche Cobas 400, Mannheim, Germany) was used to assess HbA1c.

The serum resistin and adiponectin levels were measured using a commercial enzyme-linked immunosorbent assay (ELISA) kit (Elabscience Biotechnology Inc., USA) in compliance with the manufacturer's instructions. The adiponectin-resistin (A/R) index is calculated by the equation [15]:

AR index = 1+ log10(R0)-log10(A0)

An ultrasonographic examination was performed on fasting patients after an overnight fast by the Siemens ultrasound system (Acuson S2000; ultrasound probe 4C1). During preparation for abdominal ultrasonography, patients were interviewed regarding the following data: age, gender, height and weight, alcohol consumption, history of liver disease, and intake of medications known to cause liver steatosis. Liver imaging utilizes subcostal and intercostal approaches. Each patient's ultrasound visualized the right kidney and a portion of the right liver parenchyma, which were then used for hepato-renal echogenicity ratio analysis. Ultrasound imaging indicated hepatic steatosis as more echogenic (brighter) than the renal cortex on a grayscale [16].

Data analysis

For the statistical analysis, SPSS Statistics version 22.0 was used (IBM Corp., Released 2013. IBM SPSS Statistics for Windows, Version 22.0. Armonk, NY: IBM Corp.). Continuous variables with a normal distribution were expressed as mean ± standard deviation, and continuous variables whose distribution was not normal were expressed as median (interquartile range). The Kolmogorov-Smirnov and Shapiro-Wilk tests were used to assess the distribution of variables. A two-sample t-test was used to compare continuous variables with a normal distribution, and a Mann-Whitney U test was utilized for continuous variables whose distribution was not normal. By using receiver operating characteristic (ROC) curves and the corresponding areas under the curve (AUC), the optimal biomarker cut-off values for differentiating MASLD patients from non-MASLD patients were determined. With a 95% confidence interval (95% CI), the accuracy rate for ROC curves was determined. An examination of logistic regression was done to predict when MASLD would start in patients with T2DM. A statistically significant value was defined as p<0.05.

## Results

The average values of biochemical and anthropometric parameters in T2DM individuals at the beginning of the study are shown in Table 1.

**Table 1:** Baseline characteristics of T2DM patients T2DM: type 2 diabetes mellitus; MASLD: metabolic dysfunction-associated steatotic liver disease; BMI: body mass index; HC: hip circumference; WC: waist circumference; WHR: waist-to-hip ratio; WHtR: waist-to-height ratio; HbA1c: glycated hemoglobin; TG: triglycerides; HDL-C: high-density lipoprotein cholesterol; SAP: systolic arterial blood pressure; DAP: diastolic arterial blood pressure; A/R: adiponectin/resistin

Variables	
BMI (kg/m^2^)	24.03 (23.50-24.45)
WC (cm)	90.19±8.58
HC (cm)	92.36±9.33
WHR	0.97 (0.95-1.0)
WHtR	0.54 (0.52-0.56)
Fasting glucose (mmol/L)	5.3±0.52
HbA1c (%)	5.85 (5.62-6.0)
TG (mmol/L)	1.5 (1.2-1.6)
HDL-C (mmol/L)	1.4 (1.3-1.5)
SAP (mmHg)	120.0 (120.0-130.0)
DAP (mmHg)	80.0 (80.0-90.0)
Adiponectin (ug/mL)	40.05±1.99
Resistin (ng/mL)	260.63 (244.95-269.23)
A/R index	2.69 (2.62-2.82)

Stratification of patients according to the MASLD development after 24 months demonstrated that the MASLD-T2DM group had significantly lower adiponectin (38.94±1.17 vs. 42.14±1.46 (p<0.001)), A/R index (2.65 (2.58-2.68) vs. 2.84 (2.81-2.87) (p<0.001)), and higher resistin (266.71 (261.15-271.81) vs. 240.12 (233.06-245.75) (p<0.001)) compared to the non-MASLD-T2DM group (Table 2).

**Table 2:** Baseline levels of adiponectin, resistin, and A/R index in T2DM patients related to MASLD development T2DM: type 2 diabetes mellitus; MASLD: metabolic dysfunction-associated steatotic liver disease; A/R: adiponectin/resistin

Variables	non-MASLD-T2DM group	MASLD-T2DM group	p-value
Adiponectin (ug/mL)	42.14±1.46	38.94±1.17	<0.001
Resistin (ng/mL)	240.12 (233.06-245.75)	266.71 (261.15-271.81)	<0.001
A/R index	2.84 (2.81-2.87)	2.65 (2.58-2.68)	<0.001

**Figure 1:**
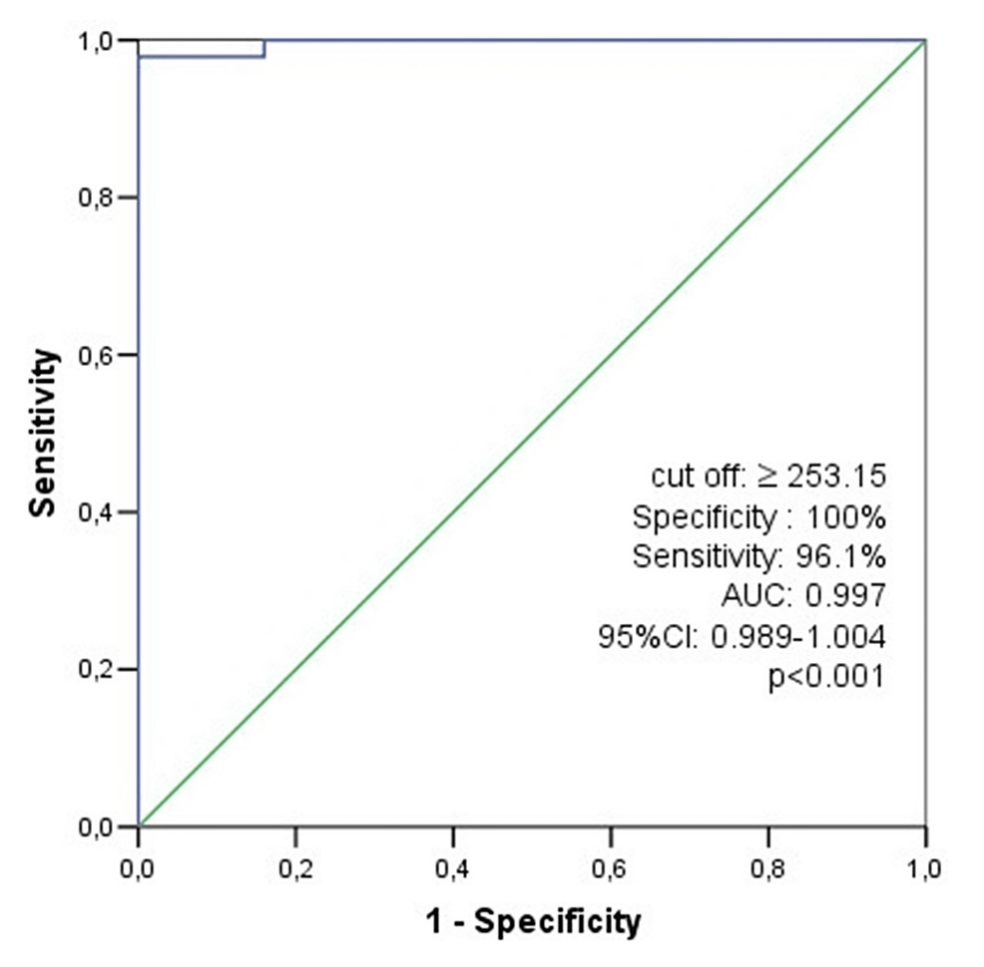
ROC curve of resistin in T2DM patients with and without MASLD ROC: receiver operating characteristic, T2DM: type 2 diabetes mellitus, MASLD: metabolic dysfunction-associated steatotic liver disease

ROC curve analyses revealed that resistin was accurate (p<0001) in predicting MASLD development (AUC of 0.997 with a sensitivity of 96.1% and specificity of 100% for the cut-off point of 253.15) (Figure 1).

Adiponectin and A/R index are not useful as a single diagnostic parameter in suspected MASLD in T2DM (0.025 (-0.013-0.062); 0.018 (-0.021-0.034)) (Table 3).

**Table 3:** AUC of adiponectin and A/R index in differentiating between T2DM patients with and without MASLD A/R: adiponectin/resistin; AUC: areas under the curve; T2DM: type 2 diabetes mellitus; MASLD: metabolic dysfunction-associated steatotic liver disease

Variables	AUC	p-value	95% CI
Adiponectin (ug/mL)	0.025	<0.001	-0.013-0.062
A/R index	0.018	<0.001	-0.021-0.034

According to the results provided by univariable logistic regression analysis, adiponectin (OR, 0.054; 95% CI, 0.011-0.268; p<0.001) and resistin (OR, 1.745; 95% CI, 1.195-2,548; p=0.004) were found to be independent predictors for MASLD development in T2DM patients (Table 4).

**Table 4:** Logistic regression analysis predicting MASLD in patients with T2DM A/R: adiponectin/resistin; MASLD: metabolic dysfunction-associated steatotic liver disease; T2DM: type 2 diabetes mellitus

Variables	Partial regression coefficient	OR (95%CI)	p-value
Adiponectin (ug/mL)	-2.928	0.054 (0.011-0.268)	<0.001
Resistin (ng/mL)	0.557	1.745 (1.195-2,548)	0.004
A/R index	-11.79	0.001 (0.000-0.002)	0.981

## Discussion

Our study aimed to assess the activity of adipocytokines in T2DM patients who will develop MASLD due to fulfilling MASLD criteria. The main finding is that adiponectin and resistin are inversely and directly related to MASLD development. In contrast, resistin responded with its sensitivity and specificity as a trusted biomarker for MASLD development in T2DM patients.

Due to recent changes in the nomenclature, MASLD covers a broad spectrum of pathological changes in liver parenchyma regarding hepatic steatosis [17,18]. Compared to previous NAFLD or MAFLD nomenclature and the fact that some patients might have average values of liver enzymes as being routinely checked in primary healthcare until ultrasound is used to spot specific parenchymal steatotic changes, the use of MASLD criteria increases the pool of T2DM patients being leaned towards metabolic illness [1,17].

Due to similarities between criteria, MASLD corresponds with metabolic syndrome (MetS) criteria, thus sharing some of the common features in the pathophysiology and clinical presentation where the IR being a cause of MetS gives off repercussions on some other human organs, such as the ovaries [1,19].

Results aligned with previous research but were termed using old nomenclature: NAFLD or MAFLD. Pan et al. [20] showed that adiponectin level was inversely related to MAFLD presence, but that relation was more substantial when only T2DM patients were analyzed as a different patient group.

Considering the literature with the old nomenclature of NAFLD, Mantovani et al. [21] showed in their observational study that the severity of the adiponectin decrease is related to the degree of steatosis in T2DM, further directly affecting the stiffness of the liver parenchyma. Contrary to the hypoadiponectinemia state, Lemoine et al. [22] suggested that adiponectin levels did not significantly differ in patients with NAFLD, although even this study included a progressive form of NAFLD, such as nonalcoholic steatohepatitis.

Regarding resistin levels in T2DM with MASLD with consideration of older nomenclature, Han et al. [23] suggested in their meta-analysis, including patients with NAFLD without stratification according to the presence of T2DM, that resistin levels were increased in the NAFLD group. However, there is still no research on resistin involvement in T2DM with MASLD.

Hyperesistinemia is usually related to T2DM and comes with MetS, where a base for high resistin is related to IR, further predisposing affected patients with a high risk for major adverse cardiac events due to proinflammatory states via cytokines such as tumor necrosis factor-alpha, interleukin-1 beta, and interleukin-6 [24].

Adiponectin’s role in MASLD pathogenesis might be related to its receptors in the way that adiponectin receptor expressions (AdipoR1 and AdiopR2) are decreased in the liver with steatotic changes simultaneously with hypoadiponectinemia, as our results showed, and therefore the adiponectin actions are mediated via activation of adiponectin receptors for further free fatty acid (FFA) oxidation [25,26]. FFA represents a potential source for the pathogenesis of MASLD since FFA levels corresponded with the development of NAFLD and its severity score, while FFA levels directly increased resistin levels, promoting IR [27,28].

A potential relation might lie in CD36 cells, whose deficiency was known to aggravate hepatic steatosis severity due to decreased FFA oxidation and, together with adiponectin, make a two-way direction in terms of potentiating their activity when it comes to the FFA oxidation process. At the same time, high resistance actively increases CD36 expression, contributing to lipid accumulation inside macrophages [29,30].

The limitations of our study included the small sample size and insufficient size, which could further stratify patients according to gender. By searching the literature, we could only find partial results to compare with our results; most studies were focused exclusively on a single disease without a causal relationship.

## Conclusions

This was a prospective study for T2DM patients in the prediction of MASLD development based on the adiponectin and resistin levels, where the resistin level in T2DM with MASLD was used for the first time. Both adiponectin and resistin were related to MASLD development in T2DM patients, while resistin was proven to be a biomarker with high sensitivity and specificity for MASLD development. Our data suggest that the adipocytokines represent predictive biomarkers to foresee the MASLD pathogenesis in T2DM patients. Based on the above, ongoing research must combine those adipokines.
